# Strengthening Health Systems for Chronic Care: Leveraging HIV Programs to Support Diabetes Services in Ethiopia and Swaziland

**DOI:** 10.1155/2012/137460

**Published:** 2012-09-27

**Authors:** Miriam Rabkin, Zenebe Melaku, Kerry Bruce, Ahmed Reja, Alison Koler, Yonathan Tadesse, Harrison Njoroge Kamiru, Lindiwe Tsabedze Sibanyoni, Wafaa El-Sadr

**Affiliations:** ^1^ICAP Columbia, Columbia University Mailman School of Public Health, 722 West 168th Street, New York, NY 10032, USA; ^2^Centers for International Programs/ICAP Ethiopia, P.O. Box 5566, Addis Ababa, Ethiopia; ^3^Pact, 1828 L Street NW, Suite 300, Washington, DC 20036, USA; ^4^Ethiopian Diabetes Association, P.O. Box 3184, Addis Ababa, Ethiopia; ^5^ICAP Lesotho, P.O. Box 13860, Maseru, Lesotho; ^6^ICAP Swaziland, P.O. Box 222 Eveni, Mbabane, Swaziland; ^7^Swaziland Ministry of Health, P.O. Box 5, Mbabane, Swaziland

## Abstract

The scale-up of HIV services in sub-Saharan Africa has catalyzed the development of highly effective chronic care systems. The strategies, systems, and tools developed to support life-long HIV care and treatment are locally owned contextually appropriate resources, many of which could be adapted to support continuity care for noncommunicable chronic diseases (NCD), such as diabetes mellitus (DM). We conducted two proof-of-concept studies to further the understanding of the status of NCD programs and the feasibility and effectiveness of adapting HIV program-related tools and systems for patients with DM. In Swaziland, a rapid assessment illustrated gaps in the approaches used to support DM services at 15 health facilities, despite the existence of chronic care systems at HIV clinics in the same hospitals, health centers, and clinics. In Ethiopia, a pilot study found similar gaps in DM services at baseline and illustrated the potential to rapidly improve the quality of care and treatment for DM by adapting HIV-specific policies, systems, and tools.

## 1. Introduction

HIV/AIDS is the leading cause of death among adults in sub-Saharan Africa (SSA), but the burden of noncommunicable chronic diseases (NCD) is high and growing [1]. The regional prevalence of diabetes mellitus (DM), for example, is expected to double between 2010 and 2030, when 28 million people in SSA are projected to be living with DM [2]. In addition to DM-specific morbidity and mortality, diabetes contributes to the burden of other noncommunicable diseases (e.g., renal and cardiovascular disease) as well as communicable diseases (e.g., pneumonia and tuberculosis), further increasing its impact on public health [3]. In 2010, 6% of total mortality in SSA was attributable to DM [4].

Unfortunately, access to prevention, care, and treatment services for NCD like DM remains out of reach for most in SSA, and health systems in lower-income countries are rarely designed to provide the continuity services required to effectively identify patients at risk, engage them in care, and retain them for the course of what is usually life-long treatment. The International Diabetes Federation estimates that 78% of those with DM in SSA remain undiagnosed [5], a consequence of limited access to trained health workers and laboratory testing as well as limited awareness of DM and its risk factors. Although there have been several promising pilot studies of nurse-led DM management and other innovations [6–9], glycemic control tends to be suboptimal for those enrolled in care, even at specialized treatment centers [10, 11]. Out-of-pocket costs for medicines, laboratory tests, and transportation create formidable barriers to adherence, as do stock-outs of drugs and supplies and the absence of effective systems to support chronic care [4, 12]. 

There is a pressing need to expand the coverage, quality, and equity of services for DM and other NCD in SSA. Although often overlooked in this context, HIV programs are the first large-scale chronic disease initiatives in the region and, as such, an important resource for those hoping to expand NCD prevention, care, and treatment. In country after country, Ministries of Health—with support from donors and partners—have developed locally owned, contextually appropriate chronic care programs for HIV. With the expansion of HIV care and treatment programs, health systems that had previously delivered only episodic acute care services have been redesigned to provide longitudinal services and lifetime care for people living with HIV (PLWH). In some cases, these changes represent innovations and new approaches, while in others they represent the availability of unprecedented levels of funding to implement time-tested strategies. 

From the health system and program management perspectives, chronic diseases have much in common with one another, whether they are communicable or noncommunicable. For example, both DM and HIV require laboratory diagnosis, daily medication (in some stages), and life-long self-management, including behavior changes. Symptoms of both diseases wax and wane over time, requiring ongoing clinical and laboratory monitoring, patient education, and adherence support. In addition, both HIV and DM may cluster within families and households, the former due to sexual and perinatal transmission and the latter due to shared genetic and environmental risk factors in some settings [4, 13]. There are also key differences, including the characteristic age groups affected, dissimilar stigma attached to the two conditions, and disease-specific mortality rates. Nonetheless, based on the key similarities, our hypothesis is that the systems, tools, and implementation strategies developed to provide continuity care for HIV in SSA can be rapidly, efficiently, and effectively utilized to support services for DM and other chronic NCD [14–17]. 

ICAP at Columbia University supports Ministries of Health and other local organizations in 21 countries, including 16 in sub-Saharan Africa. ICAP provides a wide range of HIV-related technical and infrastructure support to more than 2,500 health facilities, enabling the provision of quality comprehensive HIV/AIDS prevention, care, and treatment services. Recognizing the potential to build upon ICAP's experience to support continuity care programs for a range of chronic diseases, we embarked on two pilot studies to further the understanding of the status of NCD services and the feasibility and effectiveness of adapting HIV program-related tools and systems for patients with DM. In Swaziland, we compared systems and services for HIV and DM at 15 health facilities in order to identify opportunities for experience sharing and diffusion of innovations. In Ethiopia, a multi-component intervention adapted the approaches used in HIV clinic to enhance diabetes services at an urban referral hospital (see Table 1 for additional context). 

## 2. Materials and Methods

### 2.1. DM Program Assessment in Swaziland

In consultation with the Swaziland Ministry of Health (MOH), an analysis of NCD services was developed, using DM as an example. The study had three components: site assessments, chart review, and health care worker questionnaires. The study was approved by the MOH, and ethical approvals were obtained from the Columbia University Institutional Review Board and the Swaziland Scientific and Ethics Committee. Data were entered into a Microsoft ACCESS database, which was used for the relevant analyses.

**Site Assessments**

An existing HIV-specific site assessment tool [18] was adapted for DM, feedback was obtained from local clinicians, and the revised tool was piloted at a rural health facility. The resultant site survey tool used a checklist and rating scales to consistently and objectively describe site-level implementation of DM-specific health systems and services. Fifty-nine questions enabled the systematic description of six domains, including (1) program management; (2) organization of services and clinical care; (3) monitoring, evaluation, and medical records systems; (4) human resources; (5) laboratory capacity; and (6) pharmacy capacity. A convenience sample of 15 ICAP-supported health facilities from three of the four regions in Swaziland was selected for the survey: three of the country's six hospitals, three of five health centers, and nine of 202 health clinics. Eleven of the facilities were public and four were supported by not-for-profit nongovernmental organizations. All of the facilities had functioning HIV care and treatment clinics at the time of the site assessment. With the permission of MOH and the appropriate facility leadership, ICAP research staff completed the structured survey for each of the 15 facilities between June and August 2010, visiting relevant wards and clinics, interviewing site-level staff, and observing continuity care services for adults with DM.

**Chart Review**

A chart abstraction tool was developed to collect information from outpatient records, including clinical, laboratory, pharmacy, and counseling services recognized to be important for DM management. Only four of the 15 facilities maintained on-site medical records for outpatients with NCD, so the decision was made to abstract charts at the study site with the most available charts—Mbabane Government Hospital—recognizing that it is not representative of health facilities in Swaziland. The charts of 100 diabetic patients were sampled using a random number generator and reviewed by study staff.

**Clinician Surveys**

A questionnaire was developed to elicit the attitudes and practices of health care workers regarding programs and systems for DM. The written survey was self-administered by 72 clinicians (11 doctors, 43 nurses, 15 nurse assistants, and 3 individuals from other cadres) at three health facilities. This nonrandom sample represents approximately 17% of the physicians, 13% of the nurses, and 11% of the nursing assistants working at the three sites in 2010.

### 2.2. Pilot Study in Ethiopia

In consultation with the Federal Ministry of Health, the Ethiopian Diabetes Association (EDA), and other stakeholders, a study protocol was developed and an intervention site was selected. Ethical approvals were obtained from the Columbia University Institutional Review Board and the Oromiya Regional Health Bureau in Ethiopia. 

The study design was a single time-series (a baseline assessment, a multicomponent intervention, and a six-month follow-up assessment) conducted at Adama Hospital, an urban referral hospital 100 km southeast of Addis Ababa. At the time of the study, Adama Hospital provided HIV care to more than 17,000 adults and children, of whom 10,600 had initiated ART. Consistent with other Ethiopian health facilities, Adama Hospital provides outpatient DM services within the general outpatient department (OPD) rather than at a separate diabetes clinic.

The intervention package included strategies, systems, and tools adapted from Adama Hospital's HIV program and applied to DM services in the OPD. *Strategies* included the introduction of an “essential package” of key services, the use of step-by-step protocols, emphasis on family-focused care and point-of-service diagnosis, and identification of simple, useful monitoring and evaluation (M&E) indicators. *Systems* included appointment systems, clinical mentoring approaches, and the use of peer educators. *Tools* included appointment books, charting tools and flow sheets, job aids, and logbooks/registers. No new or experimental clinical services were introduced: all protocols were consistent with local guidelines and best practices. No additional support was provided for medications, laboratory testing, or transportation, and no new staff were engaged for implementation; services were provided by existing Adama Hospital clinicians supported by existing ICAP Ethiopia clinical advisors who also provided ongoing support for the Adama Hospital's HIV clinic.

**Baseline Assessment and Six-Month Follow-Up**

The baseline assessment, conducted in the second quarter of 2010, included systematic observation of DM services in the OPD. Patient flow was mapped, patient encounters were observed, and key informant interviews were conducted with hospital leadership, clinicians, and patients. A chart abstraction tool was developed and piloted, and the baseline chart review was conducted for the 261 adult DM patients who had visited the OPD for DM services within the prior three months. Chart review and key informant interviews were repeated six months later. Data were entered into a Microsoft ACCESS database, which was used for the relevant analyses.

**Intervention Package**

Following the approach used to support Adama Hospital's HIV clinic, an intervention package was adapted and implemented to support DM services in the OPD. Clinical equipment was procured (ophthalmoscopes, sphygmomanometers, and scales); ongoing training and clinical mentoring of nurses and doctors were provided in partnership with the EDA; HIV-specific standard operating protocols and provider support tools (wall posters, pocket guides, and desktop references) were adapted for DM, as were HIV-specific charting and health management information system tools (checklists, flow sheets, standardized forms); DM appointment books and an appointment system were introduced; a DM peer educator curriculum was developed, and training and logistical support for volunteer DM peer educators was provided; family-focused care and point-of-service DM testing for patient family members were initiated; simple standardized data were collected with ongoing feedback to clinicians.

## 3. Results

### 3.1. DM Program Assessment in Swaziland

None of the facilities utilized formal treatment algorithms or identified a basic “package of care” for DM. In contrast, HIV care and treatment were supported by national guidelines and step-by-step algorithms. In DM clinics, on-site medical records were the exception, not the rule: 4/15 (27%) of the sites had on-site medical records for DM patients, while all HIV clinics had on-site medical records and charting tools. None of the facilities had established appointment systems, defaulter tracking, adherence support programs, educational materials, or peer educators for DM, despite the fact that these services were available for PLWH. HIV clinics used structured appointment registers printed by MOH and available nationwide; the DM clinics that kept any appointment records used ad hoc handwritten notes.

Availability of equipment at DM clinics and drugs for DM patients was variable. At the time of the assessment, all the facilities had sphygmomanometers and scales, 80% had stadiometers/height measures, and only 40% had ophthalmoscopes. 100% of facilities reported that aspirin and thiazide diuretics were “usually or always” available at the pharmacy, but oral hypoglycemics were “usually or always” available at only 70% of facilities and less than half of the facilities “usually or always” had insulin, beta blockers, or ACE-inhibitors in stock. On-site access to diagnostic tests for DM was limited. Urine glucose testing was available at 93% of facilities, but blood glucose levels, creatinine, and total cholesterol tests were available at only 73%, 53%, and 26% of facilities, respectively. Two of the 15 facilities (13%) had functioning electrocardiograms on site. DM patients paid out of pocket for all medications and laboratory tests, in contrast to patients attending HIV clinic for whom drugs and diagnostic tests were provided at no cost.

**Figure 1:**
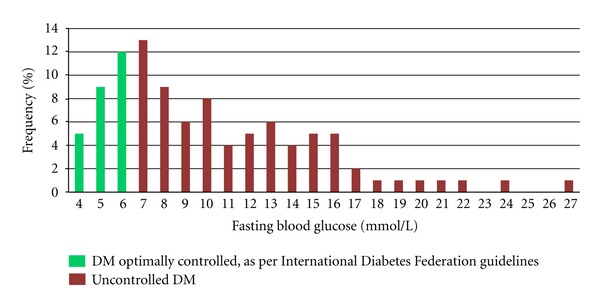
Swaziland chart review: distribution of fasting blood glucose results (*n* = 100 charts).

The median age of patients randomly selected for chart review was 56 years; 66% were female. The patients attended DM clinic fairly regularly, with 60% of patients making more than 15 documented visits since their initial enrollment. Most of the patients (81%) had been attending clinic for more than two years, and 75% had been seen at DM clinic within the past 6 months. However, only 26% of patients had optimal DM control, as defined by the International Diabetes Federation (Figure 1). In addition, 72% of the diabetic patients had HTN, defined as either a most recent blood pressure (BP) measurement of systolic BP > 140 mmHg and/or diastolic BP > 90 mmHg or at least three documented prior elevated BP measurements. Within this subgroup, only 23% were normotensive at their most recent visit. 

None of the facilities used flow sheets, checklists, or structured note templates for DM management, in contrast to many of the HIV clinics in Swaziland. At Mbabane Government Hospital, all charts had records of at least one fasting blood glucose measurement and at least one blood pressure measurement; 68% had weight documented at least once. Other documentation was scanty; current medications were documented in 1% of charts, foot exam in 7%, and fundoscopic exam in 1%. None of the charts reviewed had documentation of smoking status, medication adherence, or presence/absence of diabetic complications. 

The health care workers who completed the questionnaire indicated that barriers to provision of appropriate DM care included lack of guidelines and standard operating protocols, shortages of drugs and equipment, and inadequate staffing. Health care workers also recognized substantial barriers to adherence and retention of patients in care, including the costs of medications and laboratory tests, transportation barriers, and limited access to patient education and counseling services. Of note, 35% of respondents said that they or a family member had DM. 

### 3.2. Pilot Study in Ethiopia

The median age of DM patients at Adama Hospital was 47 years (range 18–83 years), 51% were male, 60% had type 2 DM, and 57% were currently receiving insulin. Patients had been diagnosed with DM for a median of 3 years (range 1–23 years) and had been followed at Adama Hospital for a median of 2 years (range 0–22 years). 21% had been previously hospitalized for DM, 5% had a history of foot ulcer, 20% had been diagnosed with peripheral neuropathy, 8% had reported visual impairment, and 2% had at least one amputation.

At baseline, no algorithms or standard operating protocols were used to support DM care; clinic staff reported that each individual clinician drew upon his/her training to determine optimal clinical management on a case-by-case basis. Of 45 clinicians surveyed (5 doctors, 37 nurses, and 3 other), 47% had received their degree or certification more than 10 years ago, and only 16% reported additional training in DM management since graduation. (Of note, 22% of clinicians reported having at least one family member with DM).

**Figure 2:**
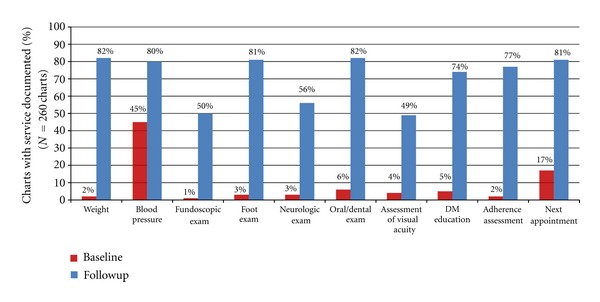
Ethiopia pilot study services offered and documented at least once in the past 3 visits**—**before and after intervention.

In the 260 charts reviewed, key clinical services were rarely documented at baseline. Although 80% of patients had a blood pressure (BP) measurement documented at least once, only 21% had ever had a documented fundoscopic exam. Foot exam had ever been documented in 10% of patients, and only 1% had a documented weight in their chart. When review was confined to the patients' three most recent visits, these rates were still lower (Figure 2).

Follow-up chart review was conducted six months after the intervention was initiated. Figure 2 shows baseline and follow-up data on documentation of services within the patients' past three visits. The percentage of charts in which patient weight was documented at least once in the past three visits increased from 2% to 82%. Documentation of BP rose from 45% to 80%. Documentation of fundoscopic exam, foot exam, and neurologic exam rose from 1% to 50%, 3% to 81%, and 3% to 56%, respectively. Adherence assessment was documented in 2% of charts prior to the intervention and 77% after the intervention; record of the next appointment date rose from 17% to 81%. Key informant interviews and focus group discussions indicated increased patient satisfaction with clinic services. 

## 4. Discussion

The program assessment identified significant gaps in the strategies, systems, and tools used to support DM services at 15 health facilities in Swaziland, as well as suboptimal control of hyperglycemia and hypertension amongst patients with DM at Mbabane Government Hospital. Although effective DM care requires longitudinal services and chronic care systems, diabetic patients in Swaziland did not have access to the types of interventions needed for appropriate management of their disease. This contrasted with the presence of functional chronic care programs developed for HIV situated within the same health care facilities. In Ethiopia, the rapid “proof-of-concept” study found similar gaps in DM services at baseline and illustrated the potential to swiftly improve the quality of care and treatment for DM by adapting HIV-specific strategies, systems, and tools. There was a marked increase in documented service delivery and significant improvements in standards of care with no added staff. 

These studies were intended to be hypothesis generating. The Ethiopia pilot study measured process indicators rather than clinical outcomes and did not include a comparison site; it is possible that alternate interventions not based on HIV programs could have achieved a similar effect. Although clinicians reported that the introduction of new systems was facilitated by the fact of local ownership—that the same systems and tools were already being used in the hospital's HIV clinic—the magnitude of this effect was not quantified. In Swaziland, site assessments and clinician surveys used nonrandom samples; the results may not be representative of health facilities and health providers nationwide. 

There are multiple ways in which to build upon the lessons and resources of HIV programs when working to expand and enhance DM and NCD services, and no single approach is likely to work in all settings. In some countries, integration of chronic disease services for HIV and NCDs at the point of care may be an effective approach [18]. In other settings, as in Ethiopia, this may be neither feasible nor desirable. A continuum of approaches is available to implementers and policy makers, ranging from parallel “side-by-side” services to complete integration of chronic disease care and treatment. The intermediate approach is one in which clinical services are not merged or integrated, but the systems behind them are shared, including guidelines, training, procurement of drugs and supplies, laboratory systems, and monitoring and evaluation strategies. 

## 5. Conclusions

In summary, the pilot studies described in this paper are among the few to document the potential of leveraging lessons learned from HIV programs to support NCD care and treatment services in sub-Saharan Africa. The findings suggest that countries which have successfully scaled up HIV services have already learned profound lessons about the delivery of chronic care. Using these locally owned and contextually appropriate resources may be an efficient and effective way to “jumpstart” NCD programs and to strengthen health systems to support longitudinal services for all. No one approach will be right for all countries and contexts, and funds are needed to support larger, controlled studies to elucidate the impact and costs of using this strategy. 

## Figures and Tables

**Table 1:** Disease burden in Swaziland and Ethiopia.

	Ethiopia	Swaziland
Population (2010)^1^	82,949,541	1,186,056
Income group (World Bank classification)	Low	Lower middle
Total health expenditure/capita ($USD)^2^	$15	$156
Life expectancy at birth (2009)^2^	54 years	49 years
HIV prevalence^2^	2.1%	25.9%
DM prevalence^3^	3.45%	2.36%
Total NCD deaths (000s)^1^	161.4 (males), 176.9 (females)	2.5 (males), 2.4 (females)
Age-standardized death rate from CVD and DM (per 100,000)^1^	486.1 (males), 530.3 (females)	558.2 (males), 441.9 (females)
% of all deaths attributable to NCDs^1^	35%	28%
NCD branch/unit/department in MOH?^1^	Yes	Yes
National diabetes plan/program?^1^	No	No

^1^WHO. Noncommunicable Disease Country Profiles 2011. Available at: http://www.who.int/nmh/publications/ncd_profiles2011/en/index.html (Accessed 8 March 2012).

^2^WHO Global Health Observatory. Available at: http://apps.who.int/ghodata/ (Accessed 8 March 2012).

^3^International Diabetes Federation. Diabetes Atlas 5th edition. International Diabetes Federation. Brussels; 2011. Available at http://www.idf.org/diabetesatlas (Accessed 8 March 2012).
